# Carcinome basocellulaire kystique à localisation bilatérale chez un albinos: à propos d’un cas

**DOI:** 10.11604/pamj.2019.34.43.19457

**Published:** 2019-09-24

**Authors:** Manix Ilunga Banza, Israël Badypwyla Tshiamala, Nathalie Dinganga Kapessa

**Affiliations:** 1 Département de Chirurgie, Faculté de Médecine, Université de Lubumbashi, Cliniques Universitaires de Lubumbashi, Katanga, Lubumbashi, République Démocratique du Congo

**Keywords:** Carcinome basocellulaire, bilatérale, térébrant, Basal cell carcinoma, bilateral, terebrant

## Abstract

Le carcinome basocellulaire (CBC) est le cancer cutané le plus fréquent. Il apparait chez les jeunes adultes au-delà de la cinquième décennie et souvent provoqué par une exposition chronique aux rayons solaires, expliquant sa fréquente localisation faciale. Il a un développement lent et une invasion locorégionale en cas de non prise en charge précoce et ne donne presque jamais de métastase à distance. Nous présentons un cas très rare de carcinome basocellulaire kystique chez un jeune adulte de 36 ans, albinos avec une localisation bilatérale; une localisation brachiale gauche sous forme ulcéro-bourgeonnante et infiltrant évoluant depuis 7 ans, térébrante avec érosion osseuse (fracture pathologique de l'humérus) et une localisation à la charnière cervico-dorsale évoluant depuis seulement 6 mois sous forme ulcéro-bourgeonnante et surinfectée. 6 poches de transfusions de sang de 450cc isogroupe et isorhésus lui ont été administrées durant son séjour de 1 mois dans notre institution hospitalière et des pansements à l'eau boriquée ont été appliqués en complément d'une antibiothérapie et d'un traitement martial. Le mauvais état général du patient avec une cachexie, une anémie chronique, l'évolution des lésions cancéreuses contrindiquaient une amputation du membre supérieur gauche et l'exérèse de la masse cervico-dorsale. En conseil pluridisciplinaire, nous avons opté pour un traitement de deuxième ligne à savoir la chimiothérapie et/ou la radiothérapie. Notre patient a ainsi été transféré à Lusaka faute d'un plateau technique suffisant pour le dit traitement.

## Abstract (en)

Basal cell carcinoma is the most common skin cancer. It occurs in young adults older than 50 years and it is often caused by chronic exposure to solar rays, which explains why it often affects the face. In patient who are not treated immediately, the tumour develops slowly, with locoregional extension, but it rarely metastasizes. We here report a very rare case of bilateral cystic basal cell carcinoma in an albino young adult aged 36 years. The patient had terebrant left brachial ulcero-budding infiltrating mass with bone erosion (pathological fracture of the humerus) evolving over the last 7 years as well as ulcero-budding secondarily infected mass in the cervicodorsal junction evolving over the last 6 months. During his 1 month stay in our hospital, he underwent transfusion of 6 units of isogroup isoRh 450cc and boric acid dressings were applied as a complement to antibiotic therapy and iron therapy. Given the poor general health of the patient (cachexia, chronic anemia, development of the cancerous lesions), amputation of the left upper member and cervicodorsal mass excision were controindicated. Multidisciplinary team opted for second line treatment, including chemotherapy and/or radiotherapy. Given the lack technical equipment, the patient was transferred out to Lusaka.

## Introduction

Le carcinome basocellulaire est le cancer cutané le plus fréquent [1]. C'est la tumeur épithéliale maligne la plus répandue et représente le tiers des cancers dans les pays occidentaux et 75% à 80% des cancers cutanés en dehors du mélanome [2]. En France, l'incidence serait de 70 pour 100 000 habitants [3]. Il représente 2%-8% des cancers cutanés chez les noirs africains [4] et 12%-35% chez les noirs américains [5]. Le carcinome basocellulaire apparait le plus fréquemment à l'âge adulte, en particulier à partir de la cinquième décennie de vie [6]; il est fréquemment localisé dans les régions qui sont chroniquement exposées au soleil [1]. Trois formes anatomo-cliniques sont classiquement décrites à savoir les carcinomes basocellulaires superficiel, nodulaire et sclérodermiforme. Quoique la clinique soit orientative au diagnostic de carcinome basocellulaire, la confirmation diagnostique reste anatomopathologique. En général, le carcinome basocellulaire est une tumeur qui se développe lentement [7], au potentiel évolutif locorégional, l'apparition des métastases étant exceptionnelle [1], même à un stade avancé [8], ce qui les différencie des carcinomes épidermoïdes qui donnent des métastases [9]; c'est donc une tumeur à malignité réduite [10]. Le carcinome basocellulaire peut s'ulcérer et avoir une évolution extrême et destructrice pouvant atteindre les structures musculaires et osseuses; qualifié ainsi de forme térébrante.

La chirurgie reste la première ligne de traitement avec comme objectif principal d'une part d'obtenir une résection complète de la tumeur et d'autre part de reconstituer le defect en utilisant les techniques optimales de réparation pour le meilleur aspect esthétique [11]. Le traitement chirurgical est le seul garant de la guérison [2]. La radiothérapie est une option méconnue, non recommandée par les sociétés internationales en première intention dans les formes localisées ou de petite taille opérables mais trouve sa place dans les formes inopérables, avancées, étendues récidivants ou encore chez les patients âgés avec nombreuses comorbidités [12]. Les cas de carcinomes basocellulaires métastatiques étaient traités par la chimiothérapie, dont malheureusement, l'efficacité n'a jamais été contrôlée dans un essai clinique et reste purement palliative. Cependant, la découverte de la voie de Hedgehod dans la physiopathologie des carcinomes basocellulaires a ouvert une nouvelle stratégie avec le développement des thérapies ciblées anti-smoothened (SMO), un élément central de la voie de hedgehod permettant de l'inactiver; avec 2 molécules disponibles à la suite d'essais de phase I et II à savoir le Vismodégib (Erivedge^®^) et le Somidégib (Odomzo^®^) [3]. Dans tous les cas, il est fondamental pour la décision thérapeutique d'avoir une preuve histologique du carcinome basocellulaire et de discuter de la prise en charge en réunion de concertation pluridisciplinaire. L'objectif du présent travail est de présenter un cas rare de localisation bilatérale de carcinome basocellulaire kystique avec une évolution térébrante contre-indiquant la chirurgie.

## Patient et observation

Nous rapportons le cas d'un patient, de sexe masculin, policier de fonction, albinos, âgé de 36 ans, venu consulter aux cliniques universitaires de Lubumbashi en date du 30/10/2018 pour une solution de continuité au niveau du bras gauche et une tuméfaction au niveau du cou. La symptomatologie remonterait à 7 ans de la présente consultation (soit en octobre 2011) par l'apparition spontanée d'un nodule au niveau du bras gauche qui, progressivement, prenait de plus en plus de volume au cours des années. Le nodule s'ulcéra au bout de la quatrième année après son apparition (en juin 2015). Le patient consulta pour la première fois un centre médical de la ville seulement après l'apparition de l'ulcération du nodule bras (en juin 2015) où une exérèse de la masse suivie des pansements à la solution de Carrel a été réalisée pendant 4 mois. L'examen anatomopathologique de la masse prélevée n'avait encore jamais été réalisé. Le patient déclare que la cicatrisation complète de la plaie opératoire n'a jamais eu lieu. L'incapacité financière du malade à poursuivre les soins médicaux l'oblige à interrompre le traitement pendant 14 mois et à recourir à la médecine tradi-moderne mais sans aucun succès. La réapparition de la masse devenant ulcéreuse et beaucoup plus large le pousse à consulter à nouveau un second centre médical de la place où le pansement local à la solution de carrel a été conduit auquel une antibiothérapie fait de Ceftriaxone injectable 3 x 1 g/j a été instauré mais sans succès au bout de 6 mois de traitement. A nouveau, le traitement a été interrompu par le patient faute de moyens financiers pendant 12 mois.

L'aggravation et même l'extension de la lésion malgré le traitement le pousse à consulter à nouveau un troisième central médical où des pansements au sérum physiologique ont été réalisés pendant 6 mois et de l'Amoxicilline gélules 500 mg à raison de 3 x 1 g/j lui a été administré pendant 4 mois sans succès. L'apparition de la douleur, de l'impotence fonctionnelle du membre supérieur gauche et l'exacerbation de l'ulcération motive le patient à consulter les cliniques universitaires de Lubumbashi pour une meilleure prise en charge. Aux antécédents, c'est un patient né septième d'une fratrie de 13 enfants dont deux albinos, notre patient ainsi que le onzième de la famille. Il ne prend pas l'alcool mais se reconnait fumeur occasionnel. Aucune notion de transfusion notée, n'a pas d'antécédent chirurgical particulier. Il est marié et est père de 6 enfants tous en vie et en bonne santé apparente. Au complément d'anamnèse, le patient signale l'apparition spontanée il y a six mois d'une seconde tuméfaction à la face postérieure du cou, légèrement douloureuse, suintant; une lourdeur du membre supérieur gauche depuis environ 2 ans devenue importante depuis 6 mois. Pas de toux, pas de fièvre. A notre examen physique, l'état général est marqué par une impotence fonctionnelle du membre supérieur gauche et un amaigrissement important (poids actuel: 51 kg; dernier poids connu mentionné par le patient était 64 kg remonte à 4 ans avant).

**Figure 1:**
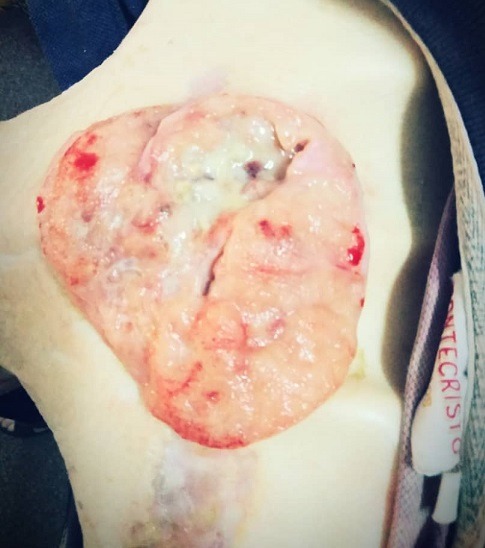
Masse ulcéro-bourgeonnante à la face postérieure du cou au niveau de la charnière cervico-dorsale

Les signes vitaux à l'admission sont 150/74 mmHg pour la tension artérielle, 110 battements par minutes pour la fréquence cardiaque, 28 cycles par minute pour la fréquence respiratoire et 36,8° pour la température. A l'examen de la tête et du cou, nous avons noté une pâleur des conjonctives palpébrales, des conjonctives bulbaires anictériques. A la face postérieure du cou, une tuméfaction médiane cervico-dorsale en regard de la sixième vertèbre cervicale à la deuxième vertèbre dorsale, bourgeonnante et ulcérée, de forme ovoïde, grand axe longitudinal 10cm et petit axe transversal 5cm, de consistance ferme, mobilisable, non adhérant au plan profond et laissant sourdre du pus, peu sensible (Figure 1). Tous les mouvements de la colonne cervicale ne sont pas perturbés. Le thorax est symétrique, de bonnes ampliations respiratoires, les bruits cardiaques réguliers. Les vibrations vocales sont bien transmises, sonorité pulmonaire normale à la percussion et à l'auscultation le murmure vésiculaire est pur. L'abdomen est non ballonné, souple, dépressible et sans aucune viscéromégalie palpée.

**Figure 2:**
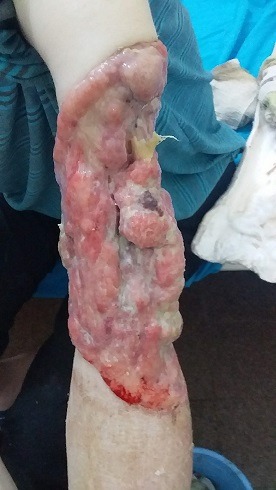
Masse ulcéro-bourgeonnante prenant le bras saignant au contact avec érosion osseuse visible traduisant une évolution térébrante de la tumeur

L'examen du membre supérieur gauche révèle une large solution de continuité ulcéro-bourgeonnante, prenant la face postéro-latérale du bras gauche, en dessous de l'épaule dont la limite supérieure est située à 10cm en dessous de l'acromion jusqu'au coude, creusant en profondeur, laissant l'humérus à nu avec une fracture localisée au tiers moyen du bras; cette solution de continuité est sensible, saignant au contact et laissant sourdre des sécrétions verdâtres (Figure 2). Pas d'adénopathie perçue ni épitrochléenne, ni axillaire. Le membre est légèrement œdématié, par rapport à celui controlatéral. L'examen vasculo-nerveux du membre supérieur gauche fait état d'une impotence fonctionnelle, d'une motricité diminuée côté à 3/5 selon la cotation de la motricité par Merle d'Aubigné, l'extension active du poignet est impossible, la sensibilité est conservée à tous les modes, le pouls radial est difficile à percevoir à cause de l’œdème mais le pouls capillaire est présent et de moins de 2 secondes, la saturation en oxygène pris au pouce est de 96%. A l'issue de l'examen clinique, les diagnostics retenus étaient: carcinome basocellulaire térébrant ou spinocellulaire du bras gauche compliqué d'infection et de fracture pathologique de l'humérus avec atteinte du nerf radial, carcinome spinocellulaire ou basocellulaire de la face postérieure du cou ulcérée et l'anémie chronique à investiguer.

**Figure 3:**
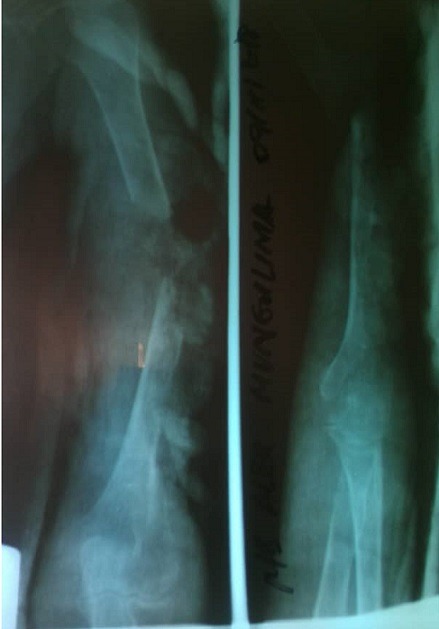
Radiographie montrant une opacité dans les parties molles avec invasion osseuse se traduisant par une perte de substance osseuse, une irrégularité des corticales

Une hospitalisation a été réalisée et des bilans paracliniques demandés que voici: la radiographie du bras gauche face et profil qui a montré une fracture de l'humérus avec une perte de substance importante (Figure 3). Un bilan d'extension fait d'une radiographie du thorax et d'une échographie abdominale a été réalisé mais était tout à fait normale. Un bilan hématologique fait d'hémoglobine Hgb (4 g%), hématocrite (14%), groupe sanguin et rhésus (O+), temps de saignement (2 minutes 30 secondes), temps de coagulation (6 minutes), le test de sérologie rapide au HIV (négative). Un typage de l'anémie avant transfusion a été réalisé et a donné comme résultat: réticulocytes 4%, volume corpusculaire moyen (MCV) 82 fL, teneur corpusculaire moyenne en hémoglobine (MCH) 30 pg, concentration corpusculaire moyenne en hémoglobine (MCHC) 330 g/l. Ensuite un bilan inflammatoire réalisé a donné comme résultat: GB: 4200/mm^3^, VS: 103 mm à la 1^ère^ heure, FL: L62% N38% M0% E1%. Il s'agissait donc d'une anémie régénérative d'origine périphérique. Quatre unités de sang de 450cc iso-groupe, iso-rhésus testés et compatible ont été administré. Une culture des secrétions verdâtre a été faite et a isolé des pyocyaniques; la solution boriquée a été indiquée pour le pansement biquotidien. La bithérapie antibiothérapique faite de Céfotaxime flacon 3 x 2 g/j et métronidazole infusion 3 x 500 mg/j a été instaurée; le tramadol ampoule 2 x 100 mg/j; le sérum anti-tétanique (3000 UI en sous-cutané après test de Besredka), le supplément martial fait de Hifer comprimé 2 x 1 cp/j. Deux prélèvements biopsiques ont été réalisés; le premier au niveau de la masse ulcéro-bourgeonnante du cou et le second au niveau de la masse ulcéro-bourgeonnante du bras avaient été réalisé en date du 19/12/2018.

Les résultats d'examen anatomopathologie ont montré une prolifération cellulaire indubitablement maligne. Les cellules proliférantes sont disposées en des amas solides avec disposition en palissade à la périphérie. Il existe également de foyers comportant de formations canalaires parfois dilatées. Le diagnostic d'un carcinome basocellulaire kystique a été ainsi retenu.

## Discussion

Le carcinome basocellulaire apparait le plus fréquemment à l'âge adulte, en particulier à partir de la cinquième décennie de vie [6]. Mohammed [10] travaillant sur une série de 45 cas de CBC de la face a trouvé un âge moyen de 66 ans avec des extrêmes allant de 37 ans et 80 ans. Notre patient est adulte mais beaucoup plus jeune que les données de la littérature, âgé de 35 ans, âge ne se trouvant même pas dans la fourchette d'âge de Mohammed ni celle de Rex Mario Razafindrakoto qui trouve des âges extrêmes de 46 et 70 ans pour une moyenne de 56,5 ans [11]; ce qui prouve que l'âge d'apparition peut être beaucoup plus précoce que celui rapporté par diverses littératures car la symptomatologie de notre patient a commencé 7 ans avant notre diagnostic soit à l'âge de 28 ans. Cette évolution lente rencontrée chez notre patient pour la première masse à localisation brachiale est cependant caractéristique du carcinome basocellulaire [7] car évoluant depuis 7 ans. La forte pigmentation cutanée des individus de race noire les protègerait contre les carcinomes cutanés; l'albinisme constituerait dans ce contexte un facteur de risque [13]. Chez les albinos, la tumeur est plus rapidement évolutive, et l'âge de survenue plus précoce que chez les patients non albinos [13], ce qui pourrait expliquer le cas de notre patient beaucoup plus jeune et l'évolution rapide de la seconde masse de la région cervico-dorsale évoluant beaucoup plus rapidement en seulement 6 mois [13, 14]. Notre patient étant de sexe masculin, cela ne nous permet pas de prédire la prédominance dudit sexe même si la majorité de littératures consultées affirme la prédominance du sexe masculin [10, 15].

Les endroits exposés chroniquement aux rayons solaires sont reconnus comme étant les sièges préférentiels de carcinome basocellulaire [11]. La tête étant la localisation la plus fréquente, le cou un peu moins et le membre encore beaucoup moins. Harkverdi *et al.* ont trouvé sur une série de 181 carcinomes basocellulaires seulement 3,04% de localisation des membres [15]. Cependant Ahmad a montré une localisation des CBC départagée de façon identique entre des zones plus exposées (front, joues et pyramide nasale) et des zones moins exposées (auricules et cuir chevelu) [16]. En plus la profession de notre patient de policier ne l'exposait pas de façon chronique aux rayons solaires contrairement aux résultats de Rex Mario Razafindrakoto chez qui ces patients étaient tous exposés au grand air par leur profession d'agriculteur, éleveur ou pisciculteur [11]. Notre patient présente une double localisation de carcinome basocellulaire aux endroits les moins rencontrés notamment la région cervico-dorsale et le bras, zones peu exposées chroniquement aux rayons solaires du fait que notre patient est policier; sa tenue de service recouvrant donc ces régions dans lesquelles siègent les CBC; ce qui est une exception par rapport aux données de la littérature qui affirment que le CBC se développerait aux endroits exposés chroniquement au soleil et rejoint par ricochet les affirmations de Ahmad [16] selon lesquelles les localisations des CBC sont départagées de façon identique entre des zones plus exposées et des zones moins exposées. Malheureusement trop peu d'articles publiés sur une localisation bilatérale ou de localisations multiples de CBC de formes histologiques différentes expliquent la pauvreté de notre discussion quant à ce. Néanmoins Karim Bourra [17] décrit une localisation bilatérale de CBC aux paupières inférieures et Moussala décrit également un autre cas de localisation faciale bilatérale [14] mais aucune publication trouvée sur une localisation bilatérale dans deux segments différents chez un même patient justifiant la particularité de notre publication.

Notre patient a consulté les cliniques universitaires 7 ans après l'apparition de la masse du bras; le retard de consultation reste un problème crucial dans notre milieu causé d'une part par la forte recrudescence des tradi-praticiens qui sont consultés en premier par la plupart des malades et d'autre part par la non vulgarisation de la politique d'un examen quasi-systématique d'anatomie pathologie pour toute masse prélevée dans nos différents centres médicaux périphériques. La non réalisation de la biopsie lors de la première exérèse nous empêche d'affirmer qu'il s'agirait d'un cas de récidive de carcinome basocellulaire. Une biopsie tumorale suivie d'un examen anatomopathologique doit être effectuée pour confirmer le diagnostic, recherchant une prolifération de kératinocytes anormaux, avec des cellules basaloïdes agencées de façon variable, en travées, en lobules ou en nodules, avec une disposition palissadique des noyaux en périphérie [18]; celui réalisé chez notre patient a révélé la forme kystique du carcinome basocellulaire qui est très peu décrit. Les CBC provoquent exceptionnellement des métastases, même à une phase avancée [11], ce qui les différencie des carcinomes épidermoïdes qui donnent des métastases. Celles-ci sont rencontrées dans 28 cas pour 10. 000 patients atteints de CBC [14]. Cependant, en l'absence de diagnostic et de chirurgie précoce, le CBC a un potentiel invasif local qui peut entrainer une destruction tissulaire importante. Le CBC peut s'ulcérer et avoir une évolution extensive et destructrice: On parlera ainsi de formes térébrantes pouvant atteindre les structures musculaires et osseuses [19]; ceci justifiera le tableau clinique de notre patient avec une forme térébrante au niveau du bras marquée par une destruction des muscles du bras, une destruction de l'humérus avec fracture pathologique médio-diaphysaire laissant l'os à nu, une atteinte du nerf radial dont nous pensons secondaire soit à une infiltration par la tumeur soit à une compression par la fracture déplacée de l'humérus (Figure 3).

Une anémie chronique type périphérique ou régénérative (réticulocytes à 4%) notée chez notre patient vivant avec un taux d'hémoglobine de 5 g/dl à l'admission serait vraisemblablement attribuable à une atteinte de la moelle osseuse siège de fabrication de globules rouges par des cellules métastatiques quoi que les métastases en cas de CBC soient exceptionnelles [20]; celles-ci sont rencontrées dans 28 cas pour 10 000 patients atteints de CBC [14]. Le traitement chirurgical des épithéliomas basocellulaires est le seul garant de la guérison [2]; la chimiothérapie n'est indiquée qu'en cas de tumeur très évoluée et inopérable [20]. Une concertation pluridisciplinaire avec l'équipe d'anatomopathologiste, d'oncologue et radiologue a résolu de surseoir le traitement chirurgical du fait des lésions avancées et d'atteinte de la lignée rouge et d'opter pour une chimiothérapie ou une radiothérapie. L'absence de molécules appropriées pour conduire la chimiothérapie et l'absence de radiothérapie dans notre pays ont motivé le transfert du patient en Zambie pour ce type de traitement qui reste d'indication en cas de lésions très avancées.

## Conclusion

Le carcinome basocellulaire est la tumeur maligne cutanée la plus fréquente. Elle est d'évolution lente, ne donne presque pas de métastases à distance mais est dotée d'un grand pouvoir d'invasion locale. La bilatéralité sur deux segments différents notamment le cou et le membre supérieur du carcinome basocellulaire kystique avec une évolution térébrante contre-indiquant tout traitement chirurgical rend encore particulier cette pathologie presque très peu décrite dans la littérature.

## Conflits d’intérêts

Les auteurs ne déclarent aucun conflit d'intérêts.
